# miR-181 interacts with signaling adaptor molecule DENN/MADD and enhances TNF-induced cell death

**DOI:** 10.1371/journal.pone.0174368

**Published:** 2017-03-21

**Authors:** Samira Ghorbani, Farideh Talebi, Sedigheh Ghasemi, Ali Jahanbazi Jahan Abad, Mohammed Vojgani, Farshid Noorbakhsh

**Affiliations:** 1 Department of Immunology, School of Medicine, Tehran University of Medical Sciences, Tehran, Iran; 2 Shefa Neuroscience Research Institute, Khatam Al-Anbia Hospital, Tehran, Iran; 3 Razi Drug Research Center, Iran University of Medical Sciences, Tehran, Iran; Indian Institute of Integrative Medicine CSIR, INDIA

## Abstract

MicroRNAs are small noncoding RNAs, which regulate the expression of protein coding transcripts through mRNA degradation or translational inhibition. Numerous reports have highlighted the role of miRNAs in regulating cell death pathways including the expression of genes involved in the induction of apoptosis. Tumor necrosis factor alpha (TNF-α) is a proinflammatory cytokine which can send pro-death signals through its receptor TNFR1. Diverse adaptor molecules including DENN/MADD adaptor protein have been shown to modulate TNF-α pro-death signaling via recruitment of MAP kinases to TNFR1 and activation of pro-survival NFκB signaling. Herein, we investigated the role of microRNA-181 (miR-181) in regulating DENN/MADD expression levels and its subsequent effects on TNF-α-induced cell death. Using bioinformatics analyses followed by luciferase reporter assays we showed that miR-181 interacts with the 3’ UTR of DENN/MADD transcripts. miR-181 overexpression also led to decreased endogenous DENN/MADD mRNA levels in L929 murine fibroblasts. Flow cytometric analysis of miR-181 transfected cells showed this miRNA accentuates mitochondrial membrane potential loss caused by TNF-α. These findings were associated with enhanced apoptosis of L929 cells following TNF-α treatment. Overall, these data point to the potential role of miR-181 in regulating TNF-α pro-death signaling, which could be of importance from pathogenesis and therapeutic perspectives in inflammatory disorders associated with tissue degeneration and cell death.

## Introduction

TNF-α is a proinflammatory cytokine, which plays critical roles in diverse inflammatory disorders [1, 2]. In addition to regulating inflammation, TNF-signaling might affect cell viability through pro-death and/or pro-survival signaling. TNFR1, a ubiquitously expressed TNF receptor, has been demonstrated to be involved in both pro-death and pro-survival TNF signaling in different cell types [3, 4]. Different studies have shown the involvement of various adaptor molecules in determining the nature of signals sent into the cells following TNF binding to TNFR1 [5, 6]. It is believed that the binding of TNFR-associated death domain (TRADD) molecule to TNFR1 leads to the recruitment of Fas-associated death domain (FADD) protein which promotes apoptosis, while the interaction with receptor-interacting protein (RIP) and TNFR-associated factor (TRAF2) can lead to cell survival. Indeed, binding of TRAF2 is believed to trigger the activation of pro-survival MAP kinases as well as the NFκB signaling [7, 8]. While TNF is widely known for its ability to induce cell death in the context of inflammation, this alternative signaling can explain the protective effects of TNF in some studies where TNF knock out or blockade has led to increased cell/tissue injury [9]. Search for mechanisms which can switch the TNF signaling from pro-apoptotic to pro-survival has led to the identification of a few adaptor molecules, including DENN/MADD (differentially expressed in normal versus neoplastic/ MAPK activating death domain). DENN/MADD is a death domain (DD)-containing protein which has been illustrated to interact with TNFR1, competing with the binding of TRADD and skewing the signaling pathway towards cell survival [10].

MicroRNAs are small non-coding RNA molecules which regulate gene expression through sequence-specific binding to target mRNAs, leading to translational silencing or transcript degradation [11]. miRNA dysregulations have been reported to influence disease process in various disorders including neurodegenerative diseases, cancers and auto immunities [12–17]. To determine the mRNA targets of each microRNA, bioinformatics together with experimental procedures have been used by researchers. miR-181 family of miRNAs is a broadly conserved group of miRNAs and its members have been revealed to influence different aspects of cell biology, including cell proliferation, differentiation and death [18–23].

**Fig 1:**
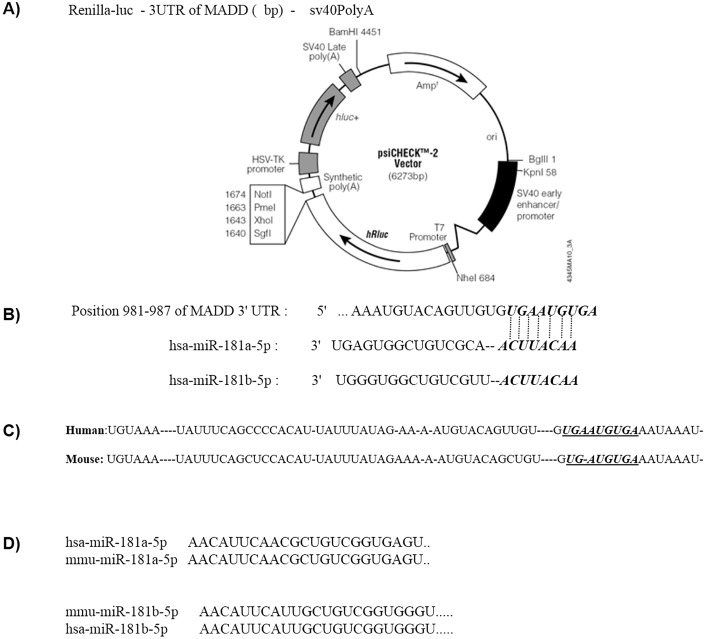
A) Luciferase reporter construct containing the 3'-UTR of human MADD mRNA. B) The miR-181a and miR-181b sequence alignments with their predicted target site in 3’UTR of MADD mRNA are presented. C). Homology between miRNA binding site at 3’UTR of MADD between human and mouse D) miR-181a and miR-181b mature miRNA sequences homology in human and mouse.

In this study, we explored the potential interactions between miR-181a or miR-181b miRNA species with DENN/MADD adaptor molecule, and their subsequent effects on TNF-mediated cell death. Bioinformatics analyses show the conserved sequence of miR-181a and miR-181b between human and mouse and a potential conserved binding site on the 3’ UTR of DENN/MADD (Fig 1). Experiments were performed to verify the interaction between miR-181a or miR-181b with the 3' UTR of DENN/MADD. miRNA transfection studies were then carried out to determine the effect of miR-181a and miR-181b overexpression on endogenous DENN/MADD levels in L929 cells, a murine fibroblast cell line which is sensitive to TNF-induced apoptosis. We next analyzed the impact of enhanced miR-181a and miR-181b expression on TNF-induced mitochondrial membrane potential alterations, Bcl2 family member expression levels and eventually cell death in L929 cells exposed to TNF-α.

## Materials and methods

### Luciferase reporter assays

Luciferase reporter assays were used to examine direct interactions between the miRNAs and the target gene. 3'UTR region of DENN/MADD was PCR amplified from genomic DNA, using primers containing appropriate restriction sites and then cloned into Psicheck vector, a Renilla luciferase reporter vector (Promega). The following primer sequences were used to amplify specific 3'UTR fragment: Forward primer: 5' TGGAGAGGGGCTACG 3', Reverse primer: 5'GGGAGTTGAGGACAT 3'. Generation of recombinant clones was confirmed by restriction digestion and sequencing.

To perform the assay, 200ng of recombinant plasmid DNA plus 100nM of microRNA mimics were co-transfected into HEK293T cells using Attractene transfection reagent (Qiagen) according to manufacturer’s instructions. HEK293T cell line was obtained from Pasture Institute of Iran (Tehran, Iran). Co-transfection with a scrambled miRNA sequence was used as a negative control. miR-181a and miR-181b mimicsand negative control were purchased from Qiagen. Luciferase activity was measured using the Dual Luciferase system (Promega) 48 hours post-transfection. Renilla luciferase levels were normalized against firefly luciferase activity as the internal transfection control. Results are presented as average ± SEM for each group.

### Cell culture

L929 (mouse fibro sarcoma cell line) was obtained from Pasture Institute of Iran (Tehran, Iran) and grown in Dulbecco’s Modified Eagle’s Medium (DMEM, Gibco) supplemented with 10% FBS (Gibco) and 100 U/ml of penicillin and 100ug/ml of streptomycin(Biosera) at 37°C. L929 cells were selected because of their sensitivity to TNF-α-induced apoptosis.

Cells were seeded in 24 well plates at a concentration of 5×10^5^ cell per well. For apoptosis induction, cells were treated with 50 ng/ml of recombinant TNF-α (eBioscience). RNA and protein extraction and flow cytometric analyses were performed after appropriate incubation times.

### miRNA transfections

miRNA-181a and 181b mimics and inhibitors as well as negative control sequence were purchased from Qiagen. Transfection assays were performed using Hiperfect transfection Reagent (Qiagen) according to manufacturer’s instructions. Briefly, 3 microliters of Hiperfect transfection Reagent was added to 100 microliters of serum free DMEM medium containing miRNA mimics or antagomirs or negative control at a final concentration of 50nM. The volume of cell culture medium in 24-well plates was adjusted to 600 microliters with medium containing 10% FBS. 24 hours post transfection; TNF-α-treated or untreated cells were harvested for RNA extraction. Transfection efficiency was assessed by expression analysis of microRNAs in transfected cells with miR-181a and miR-181b and also by monitoring green fluorescent protein (GFP) expression using fluorescence microscope.

### Quantitative real time PCR

Total RNA was extracted using GeneAll^R^ RiboEx Total RNA extraction kit (GeneAll Biotechnology) according to manufacturer’s instructions. The concentration and purity of RNA were analyzed by measuring absorbance at 260/280 nm using a nanodrop spectrophotometer (Thermo Fisher Scientific) and 1 μg of total RNA was used for cDNA synthesis.

For microRNA analyses, polyadenylation and reverse transcription was performed using miScript Reverse Transcription kit (Qiagen) which contains RT-mix, including a reverse transcriptase and a poly(A) polymerase, and the miScript RT Hispec Buffer including Mg^2+^, dNTPs, oligo-dT primers, and random primers. Reverse transcription was conducted using a thermo cycler (Applied Biosystems) with a program consisting of 37°C for 60 min and 95°C for 5 min. miRNA specific primers (miR-181a, miR-181b)were purchased from qiagen and real time PCR was carried out using miScript SYBR Green PCR Kit (Qiagen) which include universal primer. The Snord68 gene was used as an internal control for normalization. All quantitative PCR measurements were performed using a Biorad Real Time PCR system with a program consisting of 95°C for 10 min, followed by 40 cycles of 95°C for 15 s, 55°C for 30 s and 70°C for 30 s.

For gene expression analyses, reverse transcription was performed according to the protocol of PrimeScript RT Reagent Kit (Takara) using a thermo cycler (Applied Biosystems) with a program consisting of 37°C for 15 min and 85°C for 5 min. Real time PCR analysis was carried out on a Bio-Rad machine using Syber Green method. For each PCR, a master mix was prepared on ice, containing per sample: 2μl DNA, 4 μl of_5x HOT FIREPol EvaGreen qPCR Mix (Solis Bio Dyne), 0.2 mM of each reverse and forward primers. The final volume was adjusted to 20 μl with H2O. The cycling conditions were 95°C for 5 min, followed by 40 cycles of denaturation at 95°C for 30s, annealing at 55–60°C for 30 s and extension at 72C for 30 s. After amplification, a melting curve was obtained by holding the temperature at 65°C for 15 followed by heating to 95°C with a ramp rate of 0.1°C.s-1. Melt curves are shown in S1 Fig. Primer sequences used for real-time PCR are shown in Table 1. The threshold cycles were normalized to actin and the relative expression levels were calculated using 2^-ΔΔct^ method [24].

**Table 1:** Primer sets used in this study.

		Primers(5'->3')
**Gene name**	DENN/MADD (NM_001177719.1)	Forward: TAGCTTTCCAAGCTGGCTCC
		Reverse: ACCACTTTGGCTTGTCACCA
	BCL2-AssociatedX Protein(Bax) (NM_007527.3)	Forward:GTTTCATCCAGGATCGAGCAG
		Reverse: CCCCAGTTGAAGTTGCCATC
	B-cell CLL/lymphoma2 (Bcl-2)(NM_009741.5)	Forward: CCACCTGTGGTCCATCTGAC
		Reverse: CAATCCTCCCCCAGTTCACC

### Enzyme Linked Immunosorbent Assay (ELISA)

Total protein was extracted from cultured cells by adding cell lysis buffer containing 0.1 M NACL, 0.01 M tris, 0.1 mM EDTA and protease inhibitor. Protein concentration of cell lysate was determined using the standard Bradford assay. Equal amounts of protein (50Mg) from different cells were used to measure DENN/MADD level with Mouse MAP kinase-activating death domain protein, MADD ELISA Kit (Mybiosource). All steps were carried out according to the manufacturer’s instructions.

### Analysis of mitochondrial membrane potential

Loss of mitochondrial membrane potential in L929 cells was analyzed using TetraMethyl Rhodamine Ethyl Ester (TMRE). TMRE is a cell permeable, positively charged, red-orange dye that is collected in active mitochondria due to their negative charge. Cells were first transfected with 50nM of oligonucleotides (including miR-181a and miR-181b mimics, anti-miR-181a and anti-miR-181b, negative control for 24 hours and then exposed to TNF-α (50ng/ml). Following TNF-α treatment for 3 hours, TMRE (sigma) were added to the cells in media to a final concentration 100nM for 20 minutes at 37°C. Flow cytometric analysis was then performed using FACS Calibur (Becton Dickinson).

### Analysis of apoptosis

Apoptosis was induced with 50ng/ml of TNF-α and was assessed by flow cytometric analysis after 12 hours. Cells were stained by fluorescein isothiocyanate (FITC)-conjugated annexin-V and the fluorescent dye propidium iodide (PI) using the Annexin V-FITC /PI-labeled Apoptosis Detection Kit (Biolegend) according to the manufacturer’s instructions. Cells were analyzed on a FACSCalibur flow cytometer (Becton Dickinson) using the CellQuest software program. PI-negative, annexin V positive cells were considered early apoptotic cells, while PI-annexin V double positive cells were considered to be at late stages of apoptosis.

### Statistical analysis

Statistical analyses were performed using SPSS version 20 and graphs were drawn using Graph Prism. ANOVA followed by appropriate post-hoc testing was performed mean comparisons. A p value below 0.05 was considered statistically significant. All data are shown as average±SEM.

## Results

### DENN/MADD transcripts are targeted by miR-181

**Fig 2:**
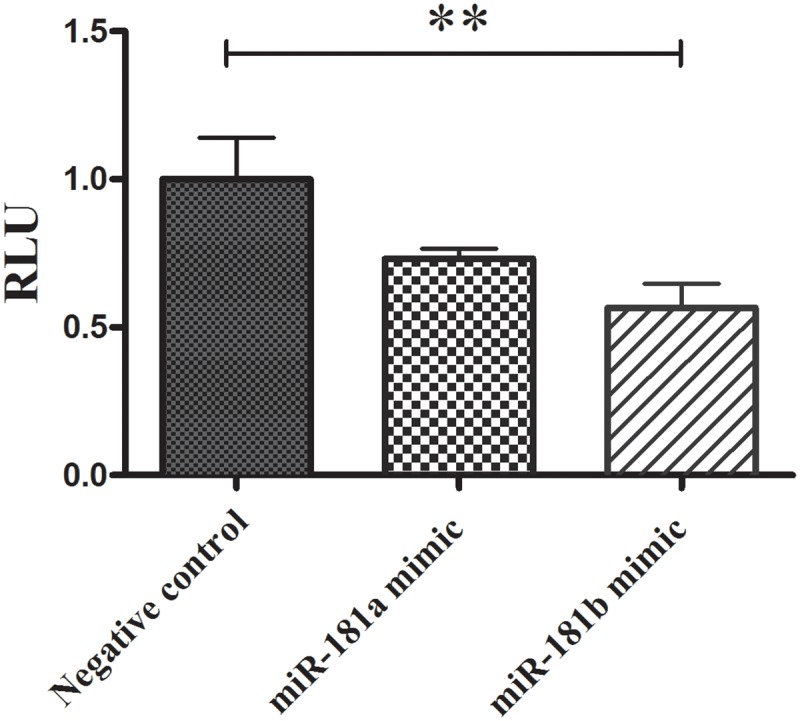
DENN/MADD is a target gene for miR-181b. Luciferase assay confirms the interaction of miR-181b with DENN/MADD transcript. Co-transfection of HEK293T cells with vectors encoding the 3'UTR of DENN/MADD ligated to the Renilla luciferase with microRNA mimic sequences and negative control sequence showed significant decrease of luciferase activity in cells transfected with miR-181 b mimic sequences. Renilla luciferase levels were normalized against internal Firefly luciferase. Data are shown as means ± SEM (*n* = 5). ANOVA, Tukey post hoc (**p≤0.01 relative to negative control)

Bioinformatics analysis using miRNA target prediction algorithms (including TargetScan) has shown that the 3’ UTR of the DENN/MADD molecule might include a potential binding site for miR-181a and miR-181b both in human and mouse. To verify the molecular interaction between the miRNA and the transcript, 3' UTR region of mouse MADD mRNA was cloned downstream of Renilla luciferase coding sequence using the Psicheck vector (Fig 1). Psicheck vector containing the 3'UTR of MADD mRNA was co-transfected along with miR-181a or miR-181b mimic sequences or negative control into HEK293T cells. Renilla luciferase activity was then measured in the lysates of the cells and normalized against the background (firefly) luciferase activity. As shown in Fig 2, Renilla luciferase activity was significantly reduced in HEK293T cells transfected with miR-181b sequence, compared with cells transfected with a scrambled miRNA sequence. These finding verify the interaction between miR-181b mature miRNA sequences with MADD’s 3'UTR, and raise the possibility that increased miRNA expression can lead to reduced MADD expression in cells.

### miR-181 repress DENN/MADD mRNA levels in L929 cells

We next studied the effect of miR-181a and miR-181b expression on endogenous mRNA expression and protein level of DENN/MADD using L929 mouse fibroblast cell line. L929 cells were selected because of their sensitivity to TNF-α-induced apoptosis. L929 cells were transfected with miR-181a or miR-181b mimics or inhibitors sequences as described in Materials and Methods. Transfection efficiency which was evaluated by fluorescence microscopy 8 h after GFP transfection in L929 cells, was around 70% (S2A Fig). Additionally, extensive increase of miR-181a and miR-181b expression levels confirmed efficiency of microRNAs over expression (S2B and S2C Fig).

**Fig 3:**
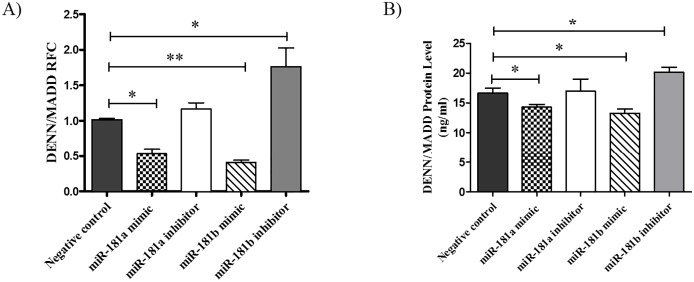
miR-181 overexpression down-regulates endogenous MADD expression. L929 cells were transfected with miR-181a, miR-181b mimics, inhibitors and negative control (50nM) for 24 or 48 hours. Cells were also treated with TNF-α (50ng/ml) for 30 minutes after transfection. mRNA expression A) protein level B) of DENN/MADD in transfected cells are shown by RT-PCR and ELISA, respectively. Data are shown as means ± SEM. (n = 3) Kruskal wallis & ANOVA, Tukey post hoc (* p<0.05, **p<0.01, relative to negative control).

DENN/MADD expression levels were analyzed at the mRNA level in L929 cells exposed to 50 ng/ml of TNF for 30 minutes after 24 hours of transfection. Real-time RT-PCR analysis showed significant reduction of DENN/MADD transcript levels in L929 cells transfected with either miR-181a or miR-181b sequences compared with cells receiving a scrambled control sequences (Fig 3A). Furthermore, miR-181b inhibitor was effective in up-regulating DENN/MADD expression.

We also examined the protein levels of DENN/MADD by ELISA after 48 hours of transfection of L929 cells with either mimic or inhibitor. Protein levels of DENN/MADD changed significantly in transfected cells, decreasing in the presence of miR-181a and miR -181b mimics and increasing after transfection with miR-181b inhibitor (Fig 3B).

These findings show that miR-181a and miR-181b can suppress the endogenous levels of DENN/MADD while only miR-181b inhibitor can increase DENN/MADD expression.

### miR-181b decreases mitochondrial membrane potential

**Fig 4:**
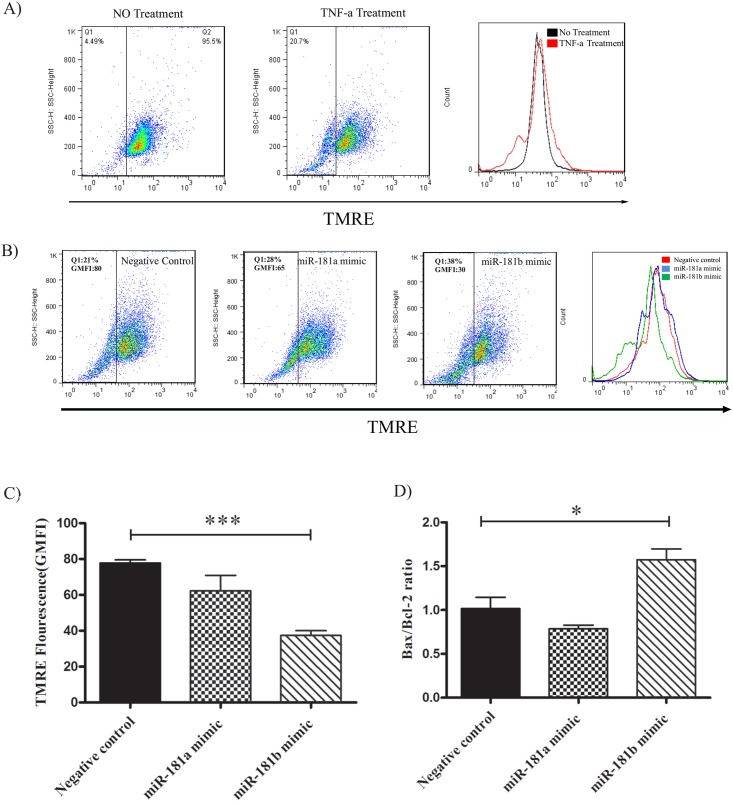
miR-181b overexpression decreases cell mitochondrial membrane potential. Following transfection of L929 cells with miR-181a, miR-181b mimics and negative control miRNA mimic (50nM) for 24 hours, cells were treated by TNF-α (50ng/ml) for 3 hours and then stained with TMRE. Quantitative analysis of Bax and Bcl-2 mRNA expression was also performed post TNF- α treatment (30 minutes) using Real time PCR A) TMRE histogram of untreated and TNF-α treated cells B) Representative flow cytometry plots of transfected cells with miRNA mimics following TNF-α exposure C) Geo mean fluorescent intensity of transfected cells with miR-181a and miR-181b mimics and negative control. D) Bax/Bcl-2 ratio following overexpression of miR-181a and miR-181b. Data are shown as means + SEM (*n* = 5). ANOVA, Tukey post hoc *p<0.05, ***p<0.000.

TNF-α is known to induce mitochondrial membrane potential loss in different cell types [25, 26]. To examine whether miR-181a or miR-181b expression might affect mitochondrial membrane potential following TNF treatment, we performed flow cytometric analysis on L929 cells transfected with miRNA or control sequences using TMRE dye, which labels active mitochondria inside the cells. As expected, treatment of L929 cells with recombinant TNF-α for 3 hours led to decreased TMRE fluorescence intensity, indicating the loss of mitochondrial membrane potential (Fig 4A). Transfection of cells with miR-181b led to further suppression of TMRE fluorescence, indicating the enhancement of TNF-induced mitochondrial changes (Fig 4B and 4C). The effect did not reach statistical significance for miR-181a. Considering the role of Bcl2 family members in regulating the mitochondrial membrane potential, we next analyzed the expression of pro-apoptotic Bax and anti-apoptotic Bcl-2 molecules following miR-181a and miR-181b transfection. Real-time PCR analysis was performed on the mRNA extracted from the miRNA transfected cells. While Bax/Bcl-2 ratio was significantly increased following miR-181b transfection, the ratio did not show a significant difference for miR-181a transfection (Fig 4D).

### miR-181 upregulation enhances TNF-induced apoptosis

**Fig 5:**
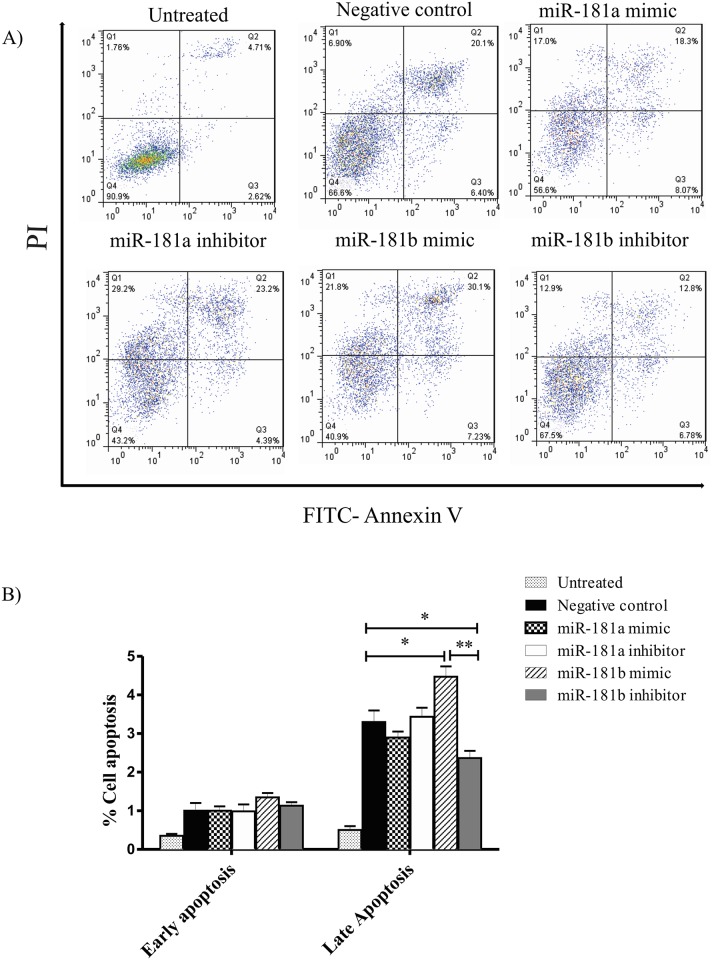
miR-181b increases TNF-α-induced apoptosis. Cultured L929 cells transfected with miR-181a and miR-181b mimics, inhibitors or negative control sequences (50nM) were treated with TNF-α (50 ng/mL) for 12 h. A) Representative flow cytometry graphs after double staining with Annexin V-FITC and propidium iodide. B) Percentage of apoptotic cells (Early apoptosis: Annexin^+^, Late apoptosis: Annexin^+^ PI^+^). Data are normalized to negative control of early apoptosis shown as means + S E M (n = 5). ANOVA, tukey post hoc *p<0.05, relative to negative control.

We finally investigated whether overexpression or inhibition of miR-181a or miR-181b might affect TNF-α induced apoptosis in L929 cells. Flow cytometric analysis of L929 cells exposed to 50 ng/ml concentration of TNF-α and stained with propidium iodide (PI) and annexin V revealed induction of apoptosis in around 30% of the cells as shown in Fig 5A. Transfection with miR-181b significantly enhanced the rate of apoptosis induced by TNF-α after 12 hours of treatment, as detected by PI/Annexin V double positive cells. On the contrary, miR-181b inhibition reduced the cell death following TNF-α treatment. Similar effect was not observed for cells transfected with miR-181a species (Fig 5B). Overall, these data point to the possibility that mir-181b overexpression can enhance cell death induced by TNF-α, an effect which might be mediated through suppression of TNF signaling adaptor molecules.

## Discussion

TNF-α in known to promote cell death and apoptosis in a variety of inflammatory conditions including cancer and neurodegenerative diseases, however, its cytoprotective effects has also been shown in other studies [9, 27–30]. While different TNF receptors might send different signals into the cells, part of the diverse effects of TNF seem to be dependent on the recruitment of the different adaptor molecules to one ubiquitously expressed receptor, i.e. TNFR1, which can switch the signaling between pro-death and pro-survival pathways [7, 8, 10]. The adaptor molecule DENN/MADD exerts an anti-apoptotic role by binding to the TNFR1 death domain. DENN/MADD can directly activate pro-survival MAP kinases or enhance the effect of TRAF2 signaling, a competitor of TRADD [10, 31, 32]. Studies regarding the role of DENN/MADD in human disease have mostly confirmed its pro-survival properties within the context of cancer. An early study by Lim et al, showed that DENN/MADD silencing by siRNAs induce apoptosis in several mammalian cancer cell lines [33]. Later studies reported the involvement of DENN/MADD in promoting cancer cell survival in the context of leukemia [34], neuroblastoma, thyroid and lung cancer [35–38]. DENN/MADDs potential role in the pathogenesis of neurodegenerative diseases has also been investigated in recent years [39, 40]. A research on neuronal cell death in Alzheimer's disease has shown significant down-regulation of DENN/MADD and enhanced hippocampal neuronal death following DENN/MADD silencing [40]. Reduced endogenous DENN/MADD expression in brain is believed to perturb neuronal survival in AD patients and transgenic mouse models of disease [39, 40]. We have also observed decreased DENN/MADD expression levels in the nervous system of EAE mice, an experimental animal model of multiple sclerosis (S3 Fig). These reports point to a potential cytoprotective effect for DENN/MADD through inhibiting TNF-induced apoptotic cell death. In the current study, we show experimental evidence pointing to the regulation of DENN/MADD by miRNA-181 through luciferase assays and transfection experiments. Among the microRNAs which are predicted to target DENN/MADD, we focused on miR-181 family members because of their implication in regulating apoptosis pathways and their broad conservation across species. Studies on human glioma and glioma cell lines have shown decreased expression of miR-181 family members together with apoptosis induction and tumor growth inhibition following miRNA overexpression [41, 42]. Other studies on astrocytes have shown increased resistance to apoptosis following miR-181 reduction likely through altered expression of Bcl-2 family members. [43]. Aberrant expression of miR-181 in neurodegenerative disease like multiple sclerosis has also been reported in previous studies [15, 17]. Gene ontology analysis on the putative targets of miR-181 also indicates the involvement of this microRNA in cellular death processes (S4 Fig).

We also provide experimental findings indicating alteration of mitochondrial membrane potential in L929 cells following miR-181 mimic transfection. In this study we used Annexin/PI assay for detection and quantification of apoptosis. This assay is able to detect externalized phosphatidyl serine molecules at the cell surface, and despite its limitations [44], has been widely used for detecting early and late stages of apoptosis. Enhanced TNF-α induced apoptosis was observed in miR-181b-transfected cells, while miR-181b antagomir sequences reduced apoptosis. Suppression of DENN/MADD could explain the resulting enhancement in TNF mediated apoptosis, however, the possibility remains that miR-181 might also affect other apoptosis mediators downstream of TNF receptor and its adaptor molecules.

Controlling signaling pathways, which affect cell viability, has immense potential in treating diseases ranging from cancers and autoimmunities to neurodegenerative disorders. In this context TNF-α signaling is of particular importance, considering the expression of this cytokine in a variety of pathophysiological settings, as well as its ability to cause both cell death and survival through the same receptors. Finding strategies to harness this ability of TNF-α and similar members of its superfamily towards enhanced cell death (e.g. in cancers) or cell survival (e.g. in neurodegenerative diseases) might provide an important tool for therapeutic interventions in human disease.

## Supporting information

S1 Fig Melting curve plots for actin, DENN/MADD, Bcl-2, Bax, Snord68, miR-181a and miR-181b genes. (TIF)

S2 Fig A) GFP-transfection efficiency was identified by fluorescence microscopy 8 h later and the image is shown. B) miR-181a, C) miR-181b expression levels after transfection with miR-181a and miR-181b mimics are shown.
(TIF)

S3 Fig DENN/MADD expression levels in mice affected by EAE. (TIF)

S4 Fig In silico analysis of target mRNAs for miR-181a, miR-181b. (TIF)
