# Supplementary figures and images for: miR-181 interacts with signaling adaptor molecule DENN/MADD and enhances TNF-induced cell death
Source: PLoS One. 2017 Mar 21;12(3):e0174368. doi: 10.1371/journal.pone.0174368 (PMC5360339; doi:10.1371/journal.pone.0174368)

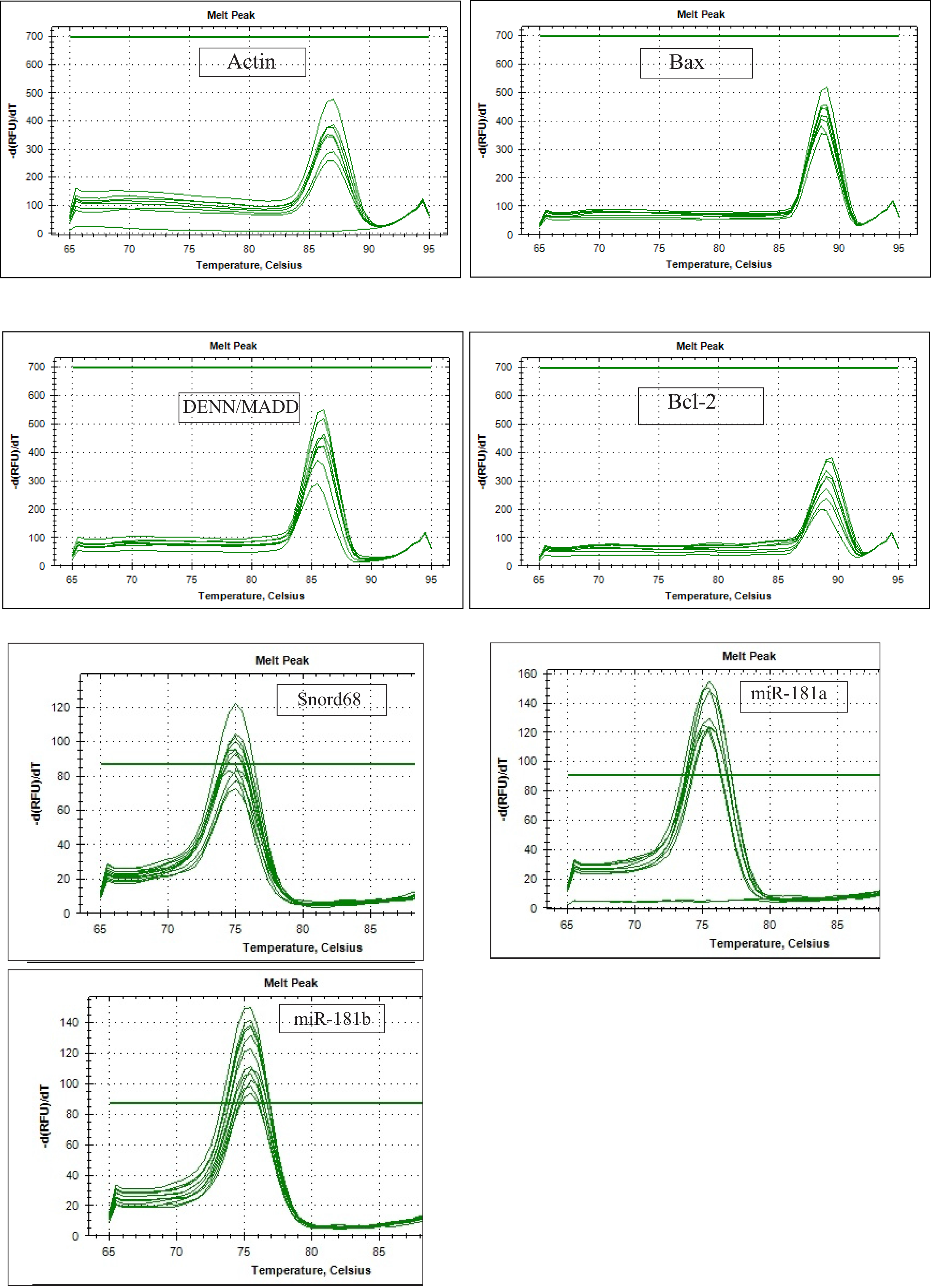

Supplement: S1 Fig — (TIF) [file pone.0174368.s001.tif]

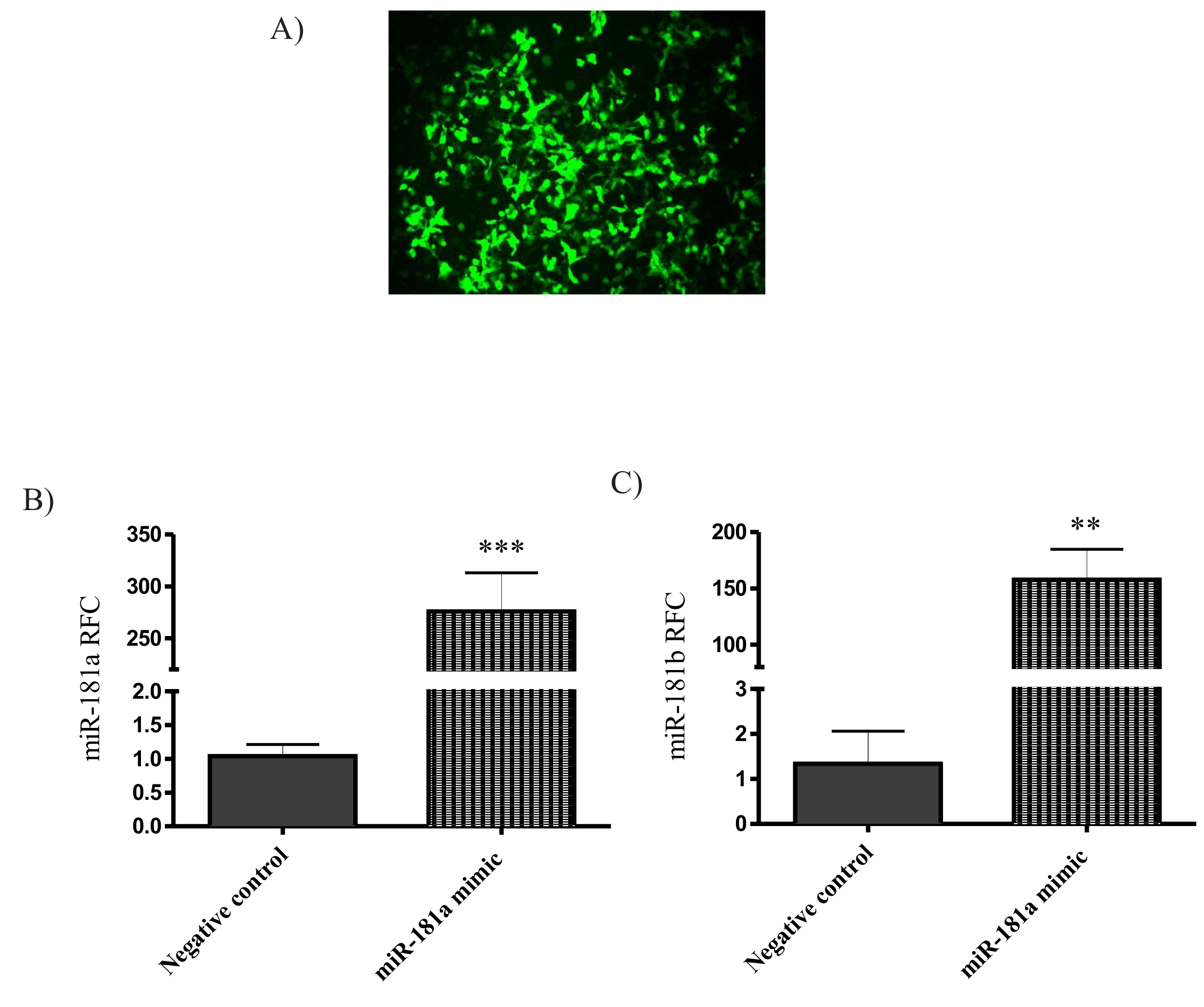

Supplement: S2 Fig — A) GFP-transfection efficiency was identified by fluorescence microscopy 8 h later and the image is shown. B) miR-181a, C) miR-181b expression levels after transfection with miR-181a and miR-181b mimics are shown. (TIF) [file pone.0174368.s002.tif]

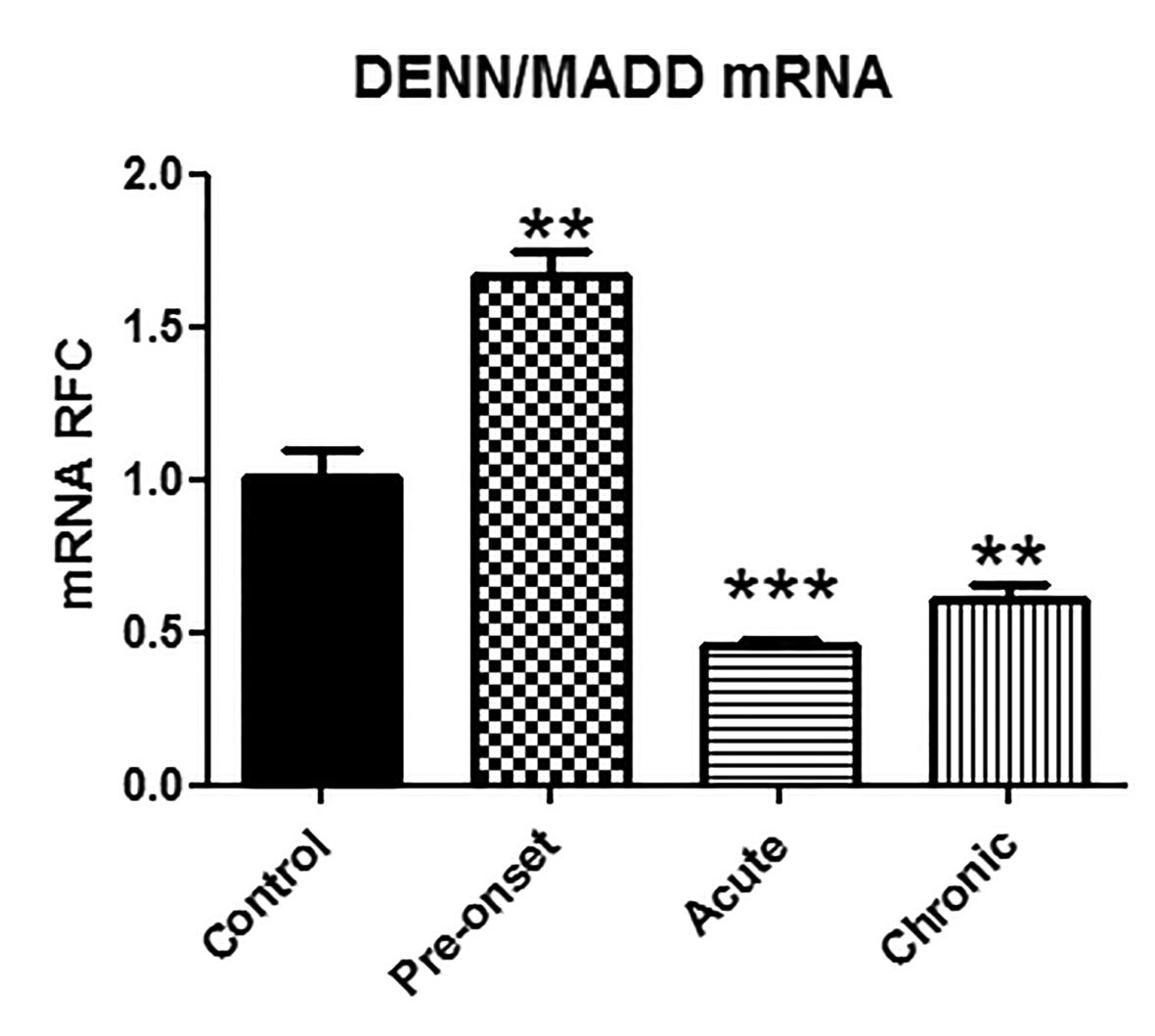

Supplement: S3 Fig — (TIF) [file pone.0174368.s003.tif]

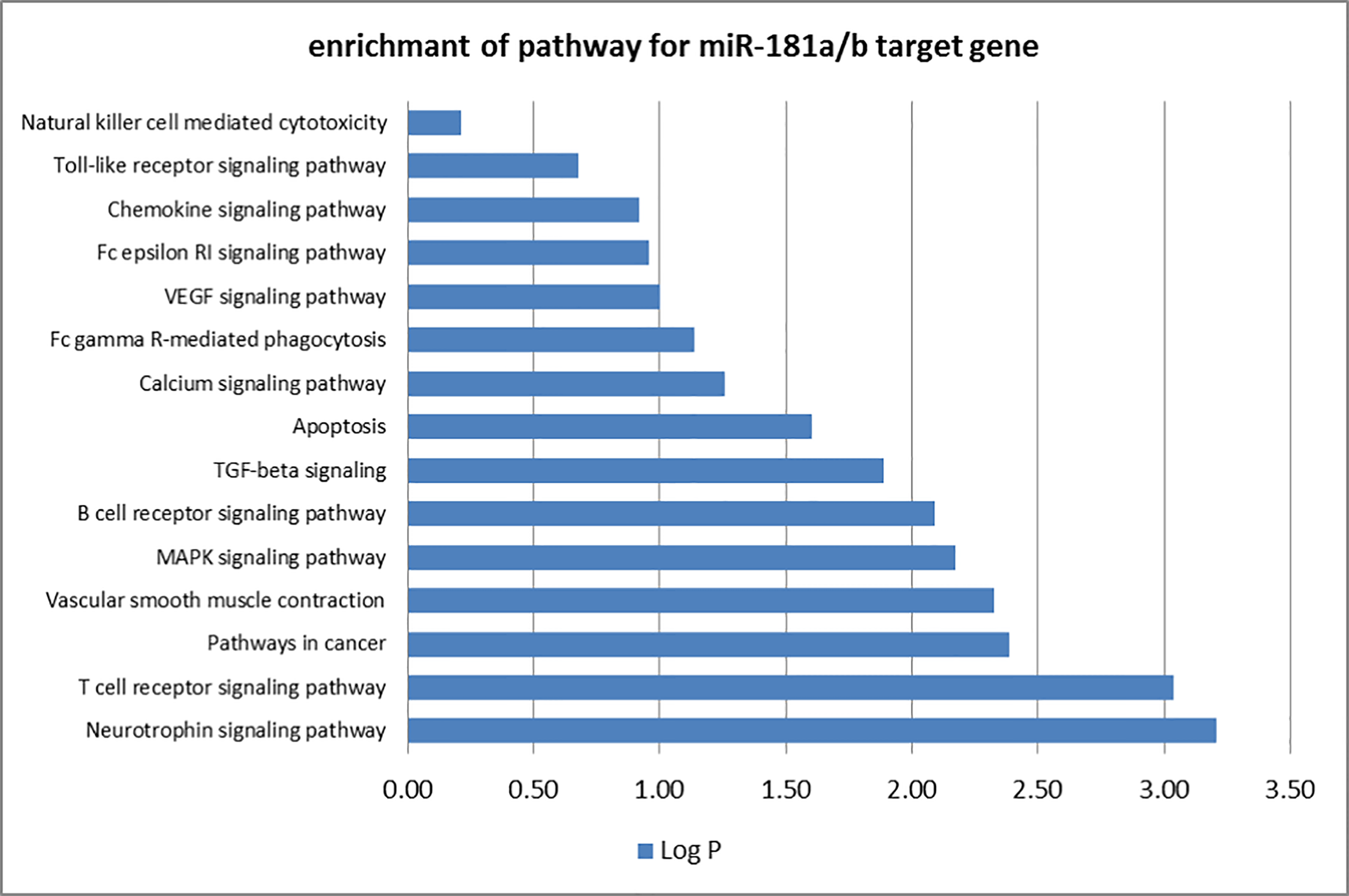

Supplement: S4 Fig — (TIF) [file pone.0174368.s004.tif]
